# Emissions of Volatile Methyl Siloxanes (VMS) from Vehicles

**DOI:** 10.1021/acs.estlett.6c00590

**Published:** 2026-07-11

**Authors:** Yanfang Chen, Yuantao Wang, Siyao Yue, Michael Bauer, Damianos Pavlidis, Carolina Molina, Georgia A. Argyropoulou, Andreas Aktypis, Christos Kaltsonoudis, Philippe Wili, Pierre Comte, Danilo Engelmann, Athanasios Nenes, Christian George, Imad El Haddad, Jay G. Slowik, Spyros N. Pandis, Andre S. H. Prevot, David M. Bell

**Affiliations:** † PSI Center for Energy and Environmental Sciences, 124377 Paul Scherrer Institute (PSI), Villigen 5232, Switzerland; ‡ Department of Chemical Engineering, 37795 University of Patras, Patras 26504, Greece; § Institute of Chemical Engineering Sciences (FORTH/ICE-HT), Patras 26504, Greece; ∥ Laboratory of Atmospheric Processes and their Impacts (LAPI), École Polytechnique Fédérale de Lausanne (EPFL), Lausanne 1015, Switzerland; ⊥ Bern University of Applied Sciences, Nidau 2560, Switzerland; # Universite Claude Bernard Lyon 1, CNRS, IRCELYON, UMR 5256, Villeurbanne F-69100, France

**Keywords:** Siloxane, Volatile Chemical Products, Vehicle Emission, Nontailpipe Emission, VOCUS-PTR-MS

## Abstract

Volatile methyl siloxanes (VMS) are anthropogenic compounds widely used in personal care products and industrial applications and are frequently detected at elevated concentrations in urban air. However, their sources in urban areas remain poorly constrained. Here, we use chassis dynamometer experiments to quantify gas-phase VMS emissions from a range of on-road vehicles, including light-duty gasoline and diesel vehicles. Hexamethylcyclotrisiloxane (D_3_) dominated the emitted VMS, over octamethylcyclotetrasiloxane (D_4_), and decamethylcyclopentasiloxane (D_5_). VMS emissions increased with driving speed and were substantially higher from gasoline than from diesel vehicles. Comparison with tunnel measurements showing elevated ambient VMS concentrations supports a significant contribution from traffic-related sources, including tailpipe and potentially nontailpipe emissions. These results identify vehicular emissions as a previously underrecognized source of VMS near highways, and provide new constraints for their atmospheric budget and source apportionment.

## Graphical abstract

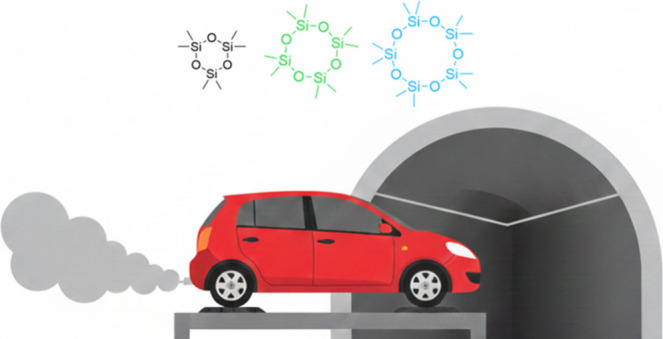

## 1 Introduction

Over the past few decades, stringent air pollution controls have shifted the dominant urban air pollution sources from fossil fuel-related traffic and industrial activities to the emissions associated with volatile chemical products (VCPs).^1,2^ As a key VCP subcategory, volatile methyl siloxanes (VMS) have drawn considerable attention due to their widespread occurrence, relative persistence and potential risks on environmental and human health.^3,4^ The main VMS compounds include octamethylcyclotetrasiloxane (D_4_, C_8_H_24_O_4_Si_4_) and decamethylcyclopentasiloxane (D_5_, C_10_H_30_O_5_Si_5_). D_4_ is commonly used as an intermediate in the production of silicone polymers.^5^ The use of D_4_ in personal care products has declined rapidly since 2002, largely due to substitution by the less hazardous D_5_.^6^ As a representative tracer for personal care products, D_5_ has been identified as the most abundant VMS in indoor environments.^7^ D_5_ typically exhibits elevated concentrations in densely populated urban areas, with levels comparable to benzene, a known traffic tracer compound.^8–10^

While the concentrations of gas-phase VMS have been extensively studied, their source attribution remains poorly constrained, particularly in urban environments where emissions from personal care products, industrial use, and potentially fossil-fuel related sources are difficult to disentangle. An et al.^11^ recently reported 13 VMS species in electric vehicle cabin air, including abundant D_4_, D_5_, and D_6_, suggesting that vehicle-associated materials and components can release substantial amounts of VMS and that vehicles may represent an underrecognized source of these compounds. Tunnel measurements have also identified methylsiloxanes in traffic-related aerosol particles and linked them to lubricant-related vehicle emissions.^12^ Extending these findings beyond tunnel environments, Yao et al.^13^ demonstrated that large molecular methylsiloxanes are widespread in atmospheric particulate matter across diverse urban, coastal, rural, and forest sites, and showed that these compounds are closely associated with traffic emissions. However, these previous studies focused primarily on particle-phase, direct measurements of speciated gas-phase VMS emissions from on-road vehicles remain lacking. Here, we identify and quantify multiple VMS species emitted by on-road vehicles and, for the first time, report emission factors for individual compounds from both tailpipe measurements and real-world traffic environments.

## 2 Materials and Methods

### 2.1 Chassis Dynamometer Measurements

Vehicle tailpipe emissions measurements were performed on a dynamometer chassis platform installed at Berne University of Applied Sciences in Biel (Switzerland). A total of 8 vehicles were tested in the dynamometer experiments, including 4 light-duty gasoline vehicles (LDGVs) and 4 light-duty diesel vehicles (LDDVs). The vehicles covered a wide range of standards spanning Euro 3 to Euro 6d with different aftertreatment technologies. Detailed vehicle information and vehicle test cycle are provided in Table S1 and Text S1. The Worldwide Harmonized Light-Duty Vehicles Test Cycle (WLTC) was used in the campaign. Trace gas emissions from the nondiluted exhaust were measured by Fourier-Transform Infrared Spectroscopy (FTIR, BOB-1000FT, A&D, Germany). In parallel, the chemical composition of nonmethane organic gases (NMOGs) was characterized by a VOCUS Proton-Transfer-Reaction-Mass-Spectrometer^14^ (VOCUS PTR-MS, Tofwerk AG, Switzerland).

### 2.2 Tunnel Measurements

Field measurements were conducted in the Fréjus road tunnel, which connects France and Italy, during May and June 2024 for a total of 3 weeks. The NMOG composition was monitored by the VOCUS PTR-MS. Trace gases were monitored by a MIRO multigas analyzer (MIRO MGA-10 GP, Analytical AG, Switzerland).^15^ The vehicle traffic in the Fréjus road tunnel was continuously monitored in both directions, with measurements distinguishing between heavy-duty vehicles (HDV) and light-duty vehicles (LDV). All air quality data inside the tunnel were corrected by subtracting ambient background concentrations measured outside the tunnel with the PSI mobile laboratory.^16^

### 2.3 Quantification of VMS

The VOCUS-PTR-MS was operated under optimized conditions with hydronium ions (H_3_O^+^) as the reagent ions. Target VMS species were identified and quantified based on their protonated molecular ions, fragment ions and clusters with water molecules.^17^ The instrument was calibrated frequently for D_3_, D_4_ and D_5_ using a certified gas standard cylinder (National Physical Laboratory, United Kingdom). The standard contained D_3_ at 910 ± 100 ppbv, D_4_ at 1150 ± 120 ppbv, and D_5_ at 1090 ± 110 ppbv, in ultrapure nitrogen. The instrumental background was regularly monitored throughout both campaigns. The details of the quantification, observed fragmentation patterns, and sensitivity of the calibrated compounds are shown in Figures S1 and S2. Fuel-based emission factors (EFs, mg kg^–1^ fuel) of VMS were calculated using a carbon-mass balance approach^18^ based on background-corrected VMS, CO_2_, and CO concentrations, as described in Text S2.

## 3 Results and Discussion

### 3.1 Dynamics of VMS Emissions from Chassis Dynamometer Experiments

Figure 1 compares selected NMOG concentrations from a light-duty gasoline vehicle (Euro 6d) and a light-duty diesel vehicle (Euro 3) during the WLTC cycle. Substantial differences are observed between the two engine types. The gasoline vehicle emits substantially higher siloxane levels than the diesel vehicle. During phases 1–3, when speeds do not exceed 97.5 km h^–1^, D_3_ and D_4_ have variable but generally low levels, while D_5_ remains largely below the detection limit. At higher speeds, particularly in the final phase, siloxane emissions from the gasoline vehicle increase sharply by at least an order of magnitude. Overall, D_3_ is the dominant VMS species, followed by D_4_ and D_5_. In contrast, siloxane emissions from the diesel vehicle remain low and show little dependence on driving conditions; only a small increase in D_3_ is observed near the end of the cycle, while the other species remain below detection. Similar temporal patterns were observed across vehicles of different emission standards (Figures S4 and S5). In contrast to the WLTC cycle, no clear siloxane emission signals were observed during idling for the same gasoline vehicle (Figure S6). Furthermore, the distinct feature of siloxane emission observed between gasoline and diesel vehicles support the interpretation that the measured siloxanes originated from vehicle-related emissions rather than contamination. The observed differences in siloxane emissions are likely attributable to higher engine temperatures of the gasoline vehicle relative to the diesel vehicle (Figure S7), which promotes the breakdown of polymeric methylsiloxanes originating from lubricating oils into lower-molecular-weight species during engine combustion. In addition to D_3_–D_5_, we also investigate linear siloxanes (L_3_–L_5_) and the higher cyclic siloxane D_6_. As shown in Figure S8, L_3_–L_5_ were not detected during the chassis dynamometer measurements for both gasoline and diesel vehicles. D_6_ was occasionally observed but remained at very low concentrations throughout the WLTC cycle, generally close to the detection limit and substantially lower than D_3_–D_5_. These observations indicate that gas-phase VMS emissions from vehicle tailpipes are dominated by D_3_–D_5_, while linear siloxanes and higher cyclic siloxanes make only minor contributions, likely because higher-molecular-weight siloxanes preferentially partition into the particle phase.

**Figure 1:**
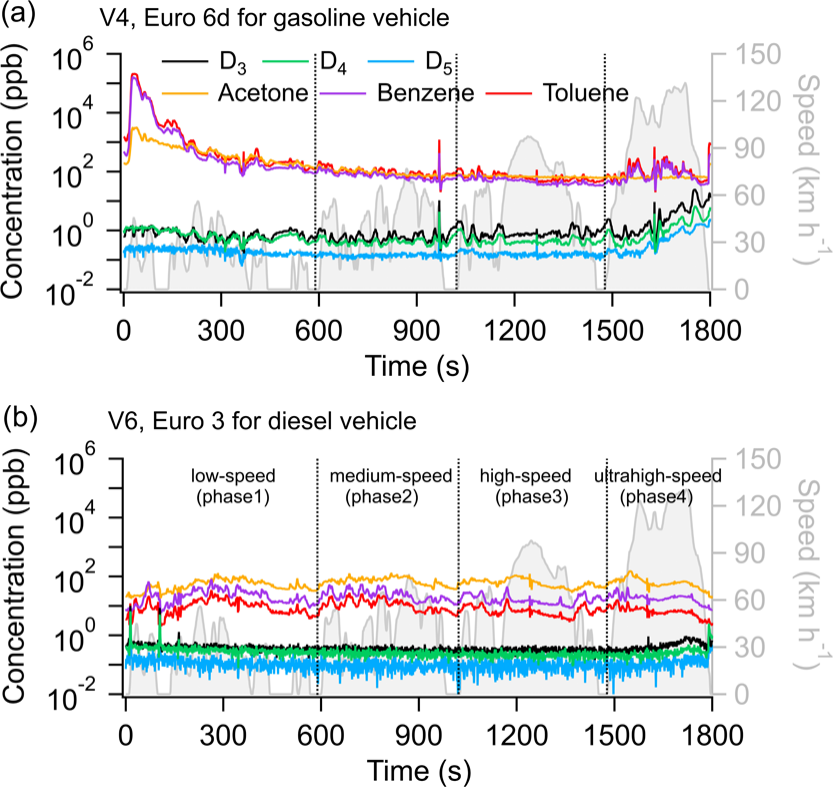
Time series of selected NMOG concentrations in the exhaust of (a) a Euro 6d light-duty gasoline vehicle and (b) a Euro 3 light-duty diesel vehicle under the cold-start conditions during the WLTC cycle. Gray shaded areas indicate the speed of the vehicles on the chassis dynamometer. The concentrations in the figures are dilution-corrected values.

The second major difference between the two vehicles is that, for the gasoline vehicle (Figure 1a), benzene and toluene peak sharply during engine start-up and decrease rapidly as the three-way catalytic converter becomes effective; acetone exhibits a similar but much weaker temporal pattern. During the extra-high-speed phase, benzene and toluene show a modest secondary increase, although concentrations remain far below low-speed phase levels. In contrast, NMOG emissions from the diesel vehicle are much lower throughout the cycle. These results confirm that cold-start conditions dominate NMOG emissions from gasoline vehicles, consistent with previous studies.^19,20^

### 3.2 VMS Emissions in the Fréjus Tunnel

Figure 2 shows in situ measurements of D_3_, D_4_, and D_5_ in the Fréjus tunnel. All three VMS species exhibited higher concentrations on weekdays than weekends, consistent with lower weekend traffic. Yao et al.^12^ have found siloxanes associated with primary organic aerosol emissions in a tunnel study, likely originating from fuel or lubricating oils. The temporal patterns of VMS differ from CO_2_ (Figure S9), suggesting that specific vehicle types or nontailpipe sources contribute to VMS emissions. To test whether D_5_ is influenced by personal care products, we examined its correlation with cashmeran (C_14_H_22_O), a personal care product tracer,^21^ and found a weak correlation (Figure S10) indicating that passenger-derived emissions were not dominant inside that environment. The D_3_/D_4_ ratio (ranging from 0.36 to 0.92, with an average of 0.69) in the tunnel (Figure 2d) is substantially lower than in tailpipe exhaust (e.g., vehicle V4:1.63 on average over the WLTC and 2.49 during phase 4), pointing to changes in emission sources under real-world traffic conditions. Evaporative emissions may contribute to vehicle-related VMS, although previous studies found siloxanes D_6_–D_9_ absent in evaporative emissions.^22^ Nontailpipe sources may also contribute to VMS in traffic-influenced environments. An et al.^11^ recently reported appreciable VMS concentrations in vehicle cabin air, suggesting that vehicle-related materials can act as VMS sources. In tunnel environments, material-related emissions and tire wear processes may therefore contribute alongside tailpipe emissions. This possibility is supported by moderate correlations (Figure S11) between VMS and benzothiazole, a tire wear tracer,^23,24^ and by the use of silicon-based materials such as silicone rubber and organosilicon coupling agents in modern tires.^25^ Together, these results suggest that real-world traffic emissions of VMS may be influenced by both tailpipe and nontailpipe sources, producing profiles that differ from direct tailpipe measurements.

**Figure 2:**
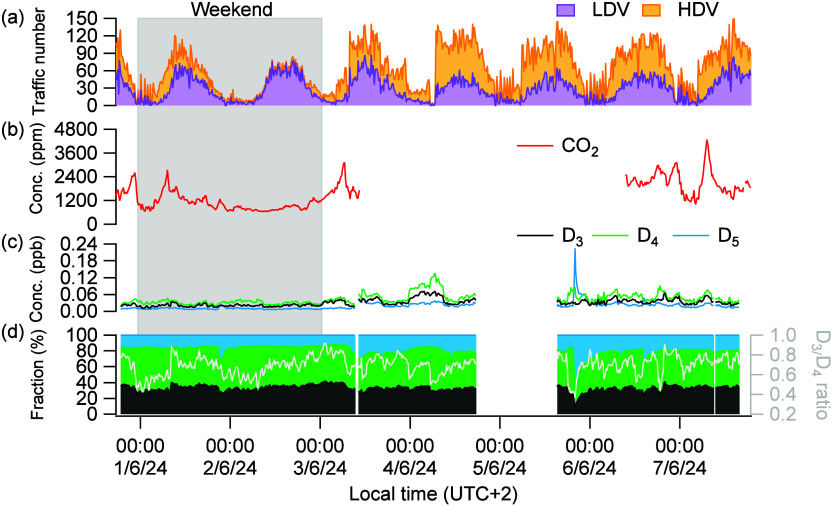
Time series of (a) traffic number, (b) trace gas, (c) representative VMS concentrations, (d) fractions and D_3_/D_4_ ratio in the tunnel.

Figure S12a shows the in situ measurements of D_6_–D_13_ in the Fréjus tunnel; the concentrations of D_6_ were very low compared with D_3_–D_5_, with the average D_4_ concentration approximately 22 times higher than that of D_6_. Other species are below the detection limits. The result is consistent with their physicochemical properties; as molecular size increases, the vapor pressure decreases.^26^ For example, at 25 °C, the vapor pressure of D_6_ (0.003 kPa) was considerably lower than those of D_3_ (0.471 kPa), D_4_ (0.121 kPa), and D_5_ (0.021 kPa). The corresponding increase in boiling point reduces the tendency of higher molecular weight siloxanes to partition into the gas phase under ambient conditions. Although D_6_ is not completely nonvolatile, their lower volatility is expected to limit atmospheric gas-phase concentrations. The D_7_–D_13_ are expected to exhibit substantially lower volatility and therefore are not expected to have a significant gas-phase fraction under ambient conditions. Instead, these compounds are more likely to partition into the particle phase, consistent with previous studies reporting the presence of high-molecular-weight siloxanes in particulate emissions from aircraft engines^27^ and traffic-related environment.^12^ L_3_–L_5_ were also not detected in the tunnel (Figure S12b), despite having vapor pressures higher than that of D_6_. In contrast, L_3_–L_5_ have previously been detected in the gas phase of emissions in residential indoor air^26^ and from hair care products,^28^ further suggesting that siloxane compositions associated with traffic emissions differ substantially from those linked to personal care product use. Taken together, these results indicate that gas-phase traffic-related VMS emissions are dominated by the more volatile cyclic siloxanes (D_3_–D_5_), whereas linear siloxanes and higher cyclic species make little contribution to the gas-phase VMS burden under the conditions investigated here.

### 3.3 Emission Factors of VMS

Fuel-based emission factors of VMS for tested vehicles are grouped into different vehicle categories. Figure 3 further demonstrates that the emissions of VMS vary significantly by fuel types, emission standard and traffic environment. The EFs of D_3_ (in the range of 0.04 to 0.65 mg kg^–1^ fuel) are generally higher than D_4_ (in the range of 0.02 to 0.30 mg kg^–1^ fuel) and D_5_ (in the range of 0.02 to 0.16 mg kg^–1^ fuel) for LDGVs and LDDVs. For LDGVs emissions, some of the variability in EFs among different vehicle categories can be attributed to the vehicle operation speed regardless of emission standards. When the driving speed shifts from high-speed (phase 3) to ultrahigh speed (phase 4), the EFs for D_3_ increase by 3.5, 4, and 5 times for the Euro 5a, 6b, and 6d vehicles, respectively. Correspondingly, the D_4_ EFs increase by 1.5, 3.3, and 3.2 times, and the D_5_ EFs increase by 1.2, 2.2, and 4 times. However, the variability within vehicles with newer emission standards suggests that VMS sources are not effectively controlled by conventional emission reduction technologies targeting criteria pollutants. EFs for LDDVs generally are lower and more consistent than those for the highest-emitting LDGVs, with modest differences across Euro standards.

**Figure 3:**
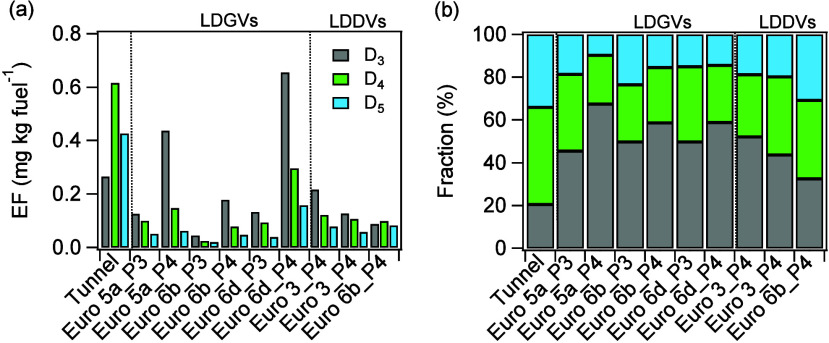
(a) Emission factors and (b) the fractions of representative VMS from the tunnel and different types of vehicles. P3 and P4 represent the high-speed phase and highway ultrahigh-speed phase during the WLTC cycle.

The tunnel measurements provide an average baseline for the real-world traffic conditions. Given that the speed limit inside the tunnel is 70 km/h, it is reasonable to compare the tunnel results with those obtained during the laboratory phase 3 driving cycle. These higher EFs of the VMS species in the tunnel imply higher emissions by the real-world fleet than those by the chassis dynamometer tests in the laboratory. Although the fractional contribution of VMS components shows similarity between LDGVs and LDDVs, the relative proportions of VMS species vary significantly from the tunnel measurements. Taken together, these results indicate that VMS concentrations in the tunnel reflect a mixed fleet average that is significantly driven by specific high-emitting vehicles and is also influenced by nontailpipe sources, as discussed in Section 3.2. Also, part of the differences could be due to the heavy-duty vehicles included in the tunnel fleet and which were not sampled during test bench measurements.

## 4 Implications

Our results demonstrate that vehicle emissions represent a previously uncharacterized source of gas-phase D_3_ and D_4_, which has not been considered in previous interpretations of atmospheric VMS measurements or in emissions inventories. This finding is particularly important for D_3_, for which appreciable urban concentrations have been reported previously but source explanations have remained limited. Previous urban measurements^29–31^ have reported appreciable D_3_ and D_4_ concentrations, but their sources were not fully resolved. The identification of vehicle emissions as a source of D_3_ and D_4_ therefore provides a plausible explanation for these previously observed urban VMS patterns. The D_4_/D_5_ ratios observed in this study, approximately 2.86 in the chassis dynamometer measurements and 2.35 in the tunnel measurements, are substantially higher than those typically observed in personal-care-product-dominated urban air, including New York City, USA (∼0.22),^10^ Shanghai, China (∼0.22),^32^ and Toronto, Canada (∼0.3).^33^ Instead, these ratios are closer to those reported for industrially influenced environments^32^ (∼1.55). Elevated D_4_/D_5_ ratios associated with industrial sources were also recently reported by Xiao et al.^34^ in Guangzhou, China. The elevated D_4_/D_5_ ratios observed here indicate that traffic-related emissions may exhibit a distinct VMS composition compared to typical urban background air. The D_3_/D_4_/D_5_ profiles provide further evidence for this distinction. We measured D_3_/D_4_/D_5_ ratios of 1.00:1.46:0.62 in tailpipe emissions and 1.00:0.52:0.18 in tunnel air, indicating that D_3_, together with D_4_, is an important component of vehicle-related VMS emissions. This profile differs from personal care product emissions, which are typically dominated by D_5_, and suggests that D_3_ and D_4_ may serve as useful indicators of traffic-related VMS contributions in urban environments.

Taking Chicago as an example, and using that the emission rate of nonvehicular D_5_ (190 mg capita^–1^ d^–1^) reported by Buser et al.,^35^ the outdoor D_5_ concentration measured in Chicago by Coggon et al.^9^ (∼10 ppt), together with a fuel consumption of approximately 6 kg capita^–1^ d^–1^ and the vehicular D_5_ emission factor (0.42 mg kg^–1^ fuel) derived from our tunnel measurements, vehicular emissions are estimated to contribute only ∼ 0.13 ppt to outdoor D_5_ concentrations. In contrast, using the D_4_ emission factor measured from tunnel (0.62 mg kg^–1^ fuel), the D_4_ per-capita emission rate used by Janechek et al.^36^ (32.8 mg capita^–1^ d^–1^), and the outdoor D_4_ concentration reported in Chicago^37^ (∼4.45 ppt), vehicular emissions are estimated to contribute ∼ 0.50 ppt, or ∼ 11% of ambient D_4_. This comparison suggests that vehicle emissions may be more relevant for interpreting ambient D_4_ than D_5_, consistent with the elevated D_4_/D_5_ ratios observed in this study.

Overall, this study advances our understanding of traffic-related VMS emissions through an integrated analysis of laboratory chassis dynamometer tests and real-world tunnel measurements. By identifying and quantifying speciated gas-phase VMS emissions from vehicles for the first time, our results reveal a previously unreported source of D_3_ and D_4_ with implications for urban air quality, source attribution, and future emissions inventories.

## Supplementary Material



## Data Availability

The data presented in this study is archived in the Zenodo repository and will be publicly available at 10.5281/zenodo.20492760 upon the publication of this article.

## Notes

Additional detailed information for tested vehicles and testing procedures, VOCUS-PTR-MS sensitivity, calculation of emission factors, NMOG emission profiles of tested vehicles not shown in the main text, exhaust gas temperature measurements, tunnel ventilation system operation and parameters, and correlation analysis among selected NMOG species measured inside the tunnel (PDF)
