# Supplementary material for: Emissions of Volatile Methyl Siloxanes (VMS) from Vehicles
Source: Environ Sci Technol Lett. 2026 Jul 11;13(8):1145–50. doi: 10.1021/acs.estlett.6c00590 (PMC13480128; doi:10.1021/acs.estlett.6c00590)
Supplement: Supplementary file 1 [file ez6c00590_si_001.pdf]

Supplementary Information for:

Emissions of Volatile Methyl Siloxanes (VMS) from Vehicles

Yanfang Chen[1], Yuantao Wang[1], Siyao Yue[1], Michael Bauer[1], Damianos Pavlidis[2, 3], Carolina Molina[3], Georgia A. Argyropoulou[2, 3], Andreas Aktypis[2, 3], Christos Kaltsonoudis[3], Philippe Wili[5], Pierre Comte[5], Danilo Engelmann[5], Athanasios Nenes[3,4], Christian George[6], Imad El Haddad[1], Jay G. Slowik[1], Spyros N. Pandis[2, 3], Andre S. H. Prevot[1*], David M. Bell[1*]

[1]PSI Center for Energy and Environmental Sciences, Paul Scherrer Institute (PSI), Villigen, 5232, Switzerland

[2]Department of Chemical Engineering, University of Patras, Patras, 26504, Greece

[3]Institute of Chemical Engineering Sciences (FORTH/ICE-HT), Patras, 26504, Greece

[4]Laboratory of Atmospheric Processes and their Impacts (LAPI), École Polytechnique Fédérale de Lausanne (EPFL), Lausanne, 1015, Switzerland

[5]Bern University of Applied Sciences, Nidau, 2560, Switzerland

[6]Universite Claude Bernard Lyon 1, CNRS, IRCELYON, UMR 5256, Villeurbanne, F-69100 France.

[*]Corresponding author, Email: andre.prevot@psi.ch, david.bell@psi.ch

For Environ. Sci. Technol. Lett

This file includes:

9 Pages

2 Text sections

12 Figures

1 Table

Text S1. Vehicle Testing Procedure

The Worldwide Harmonized Light-Duty Vehicles Test Cycle (WLTC) was used in the campaign. This cycle simulates the real-world driving conditions for passenger cars and light commercial vehicles with four operating phases: urban (low-speed of ~19 km/h, 0−589 s, phase 1), suburban (medium-speed of ~40 km/h, 590−1022 s, phase 2), rural (high-speed of ~57 km/h, 1023−1477 s, phase 3) and highway (ultrahigh-speed of ~92 km/h, 1478−1800 s, phase 4). Each vehicle was tested under cold-start conditions through a complete WLTC cycle. In this study, a cold start was defined as the first cycle following engine start at ambient temperature. The emissions were diluted with a dilution ratio of 10～400 for different vehicles before they were measured by VOCUS PTR-MS. During chassis dynamometer measurement, the sampling lines were flushed with zero air before and after each experiment to minimize potential background contamination. Gasoline and diesel vehicles were tested in a mixed order rather than sequentially by fuel type, which helped minimize the potential influence of systematic contamination or carryover effects. Details are listed in supplementary Table S1. In addition, research personnel avoided the use of personal care products during the measurement periods.

Text S2. Calculation of Emission Factors

The fuel-based emission factors (EFs, mg kg^{-1} fuel) of VMS were calculated following a carbon-mass balance approach[1].

$$EF_i = \frac{\Delta \mathrm{P}}{\Delta \mathrm{CO_2} + \Delta \mathrm{CO}}\,\omega_c \qquad (1)$$

where ΔP is the background-corrected concentration of the species of interest P, ω_c is the carbon

fraction of the fuel equal to 0.86 in our case, and ΔCO_2 and ΔCO are the corresponding background-corrected carbon oxide mass concentrations. The EFs for each type of vehicle were averaged across each of the driving phases. Measurements falling below the detection limit (LOD, defined as three times the standard deviation of the instrument background divided by the sensitivity) were excluded from the analysis. The average LODs for D_3, D_4 and D_5 are 1.11, 0.93 and 0.83 pptv respectively. To estimate the EFs in the tunnel, only the data collected while the tunnel ventilation system was operating (6:00 am–6:00 pm; Figure S3) were used.

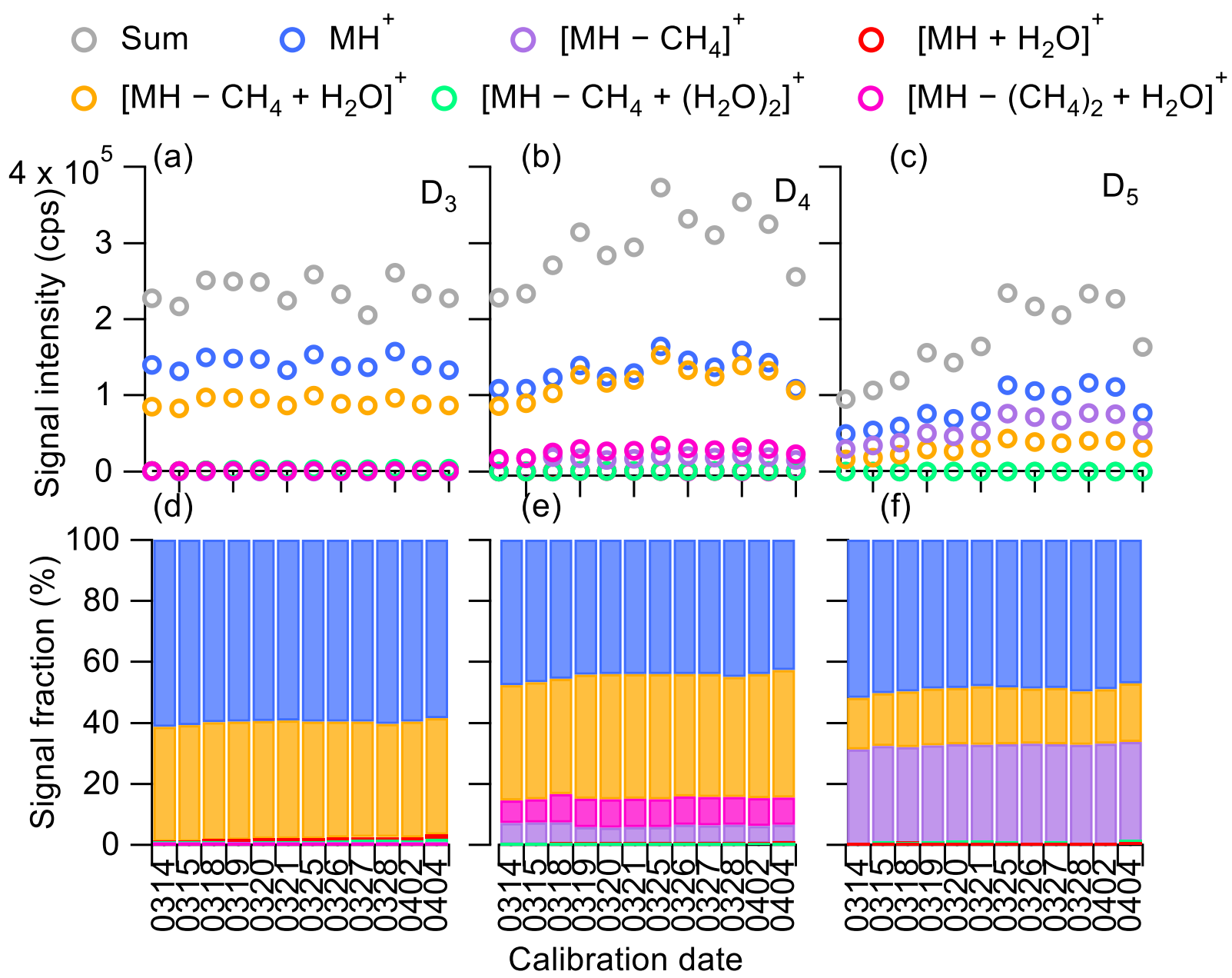

Figure S1. The signal intensities and fractions of protonated ion, adduct ions and fragmented ions for D_3, D_4 and D_5.

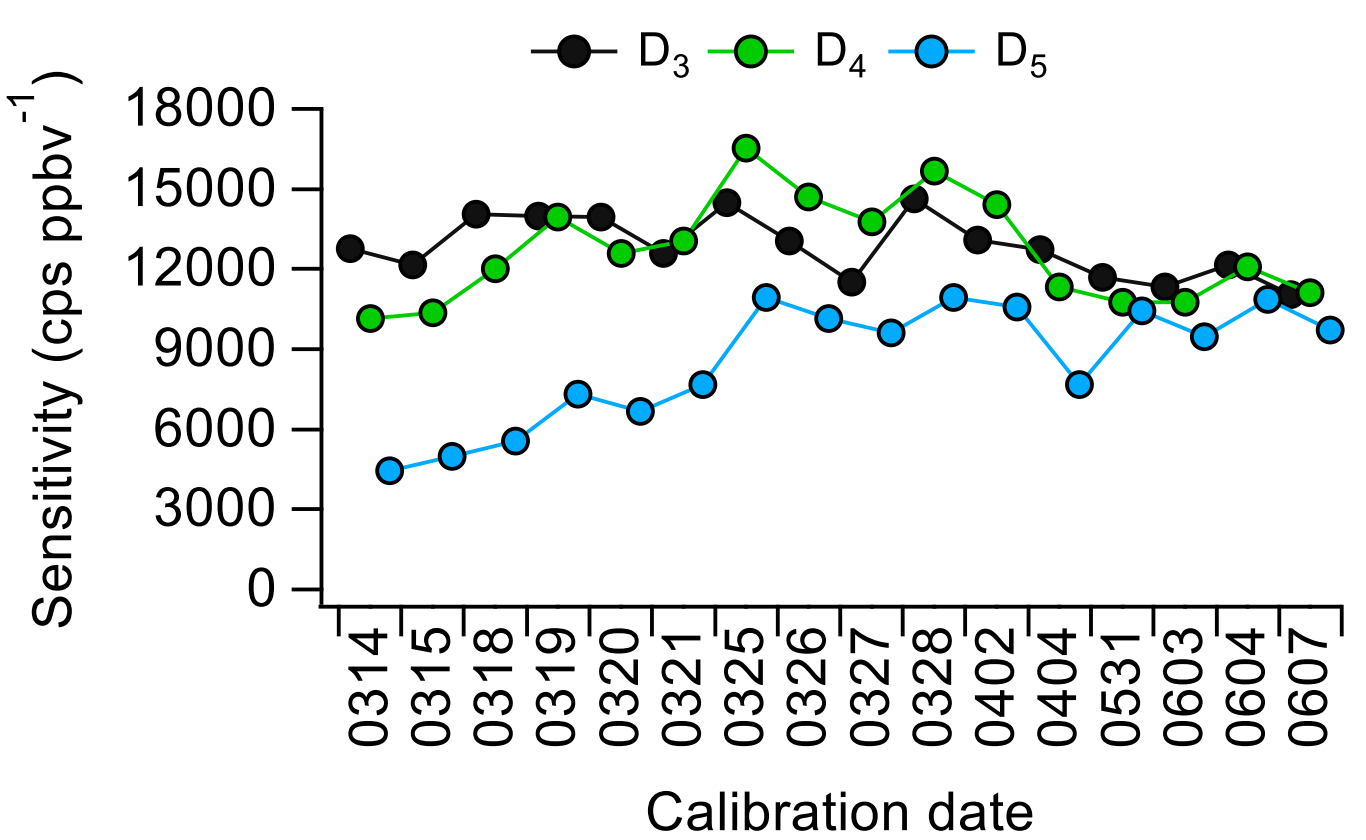

Figure S2. The VOCUS-PTR-MS sensitivity for D_3, D_4 and D_5.

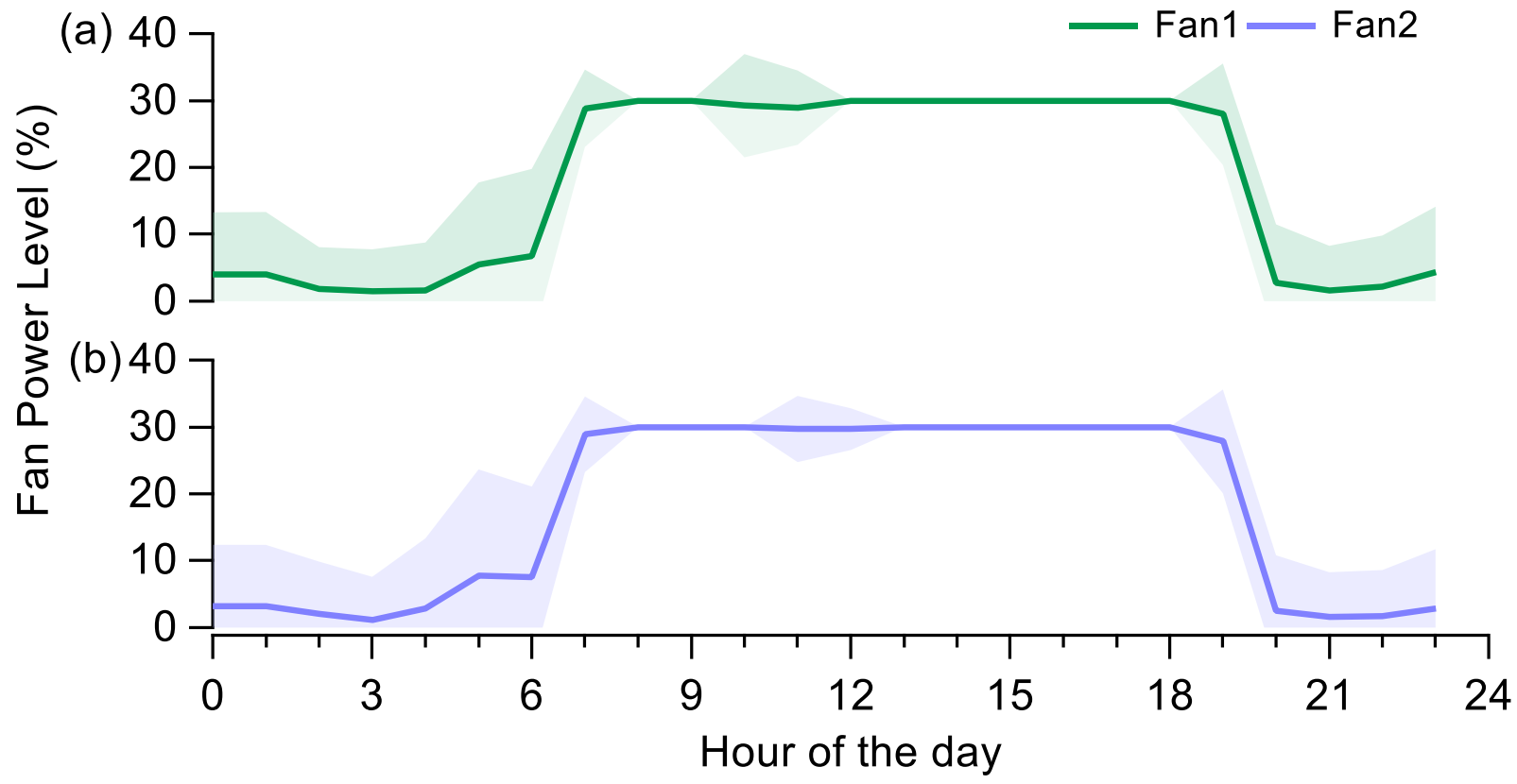

Figure S3. Operating power levels of ventilation fans near the tunnel sampling location.

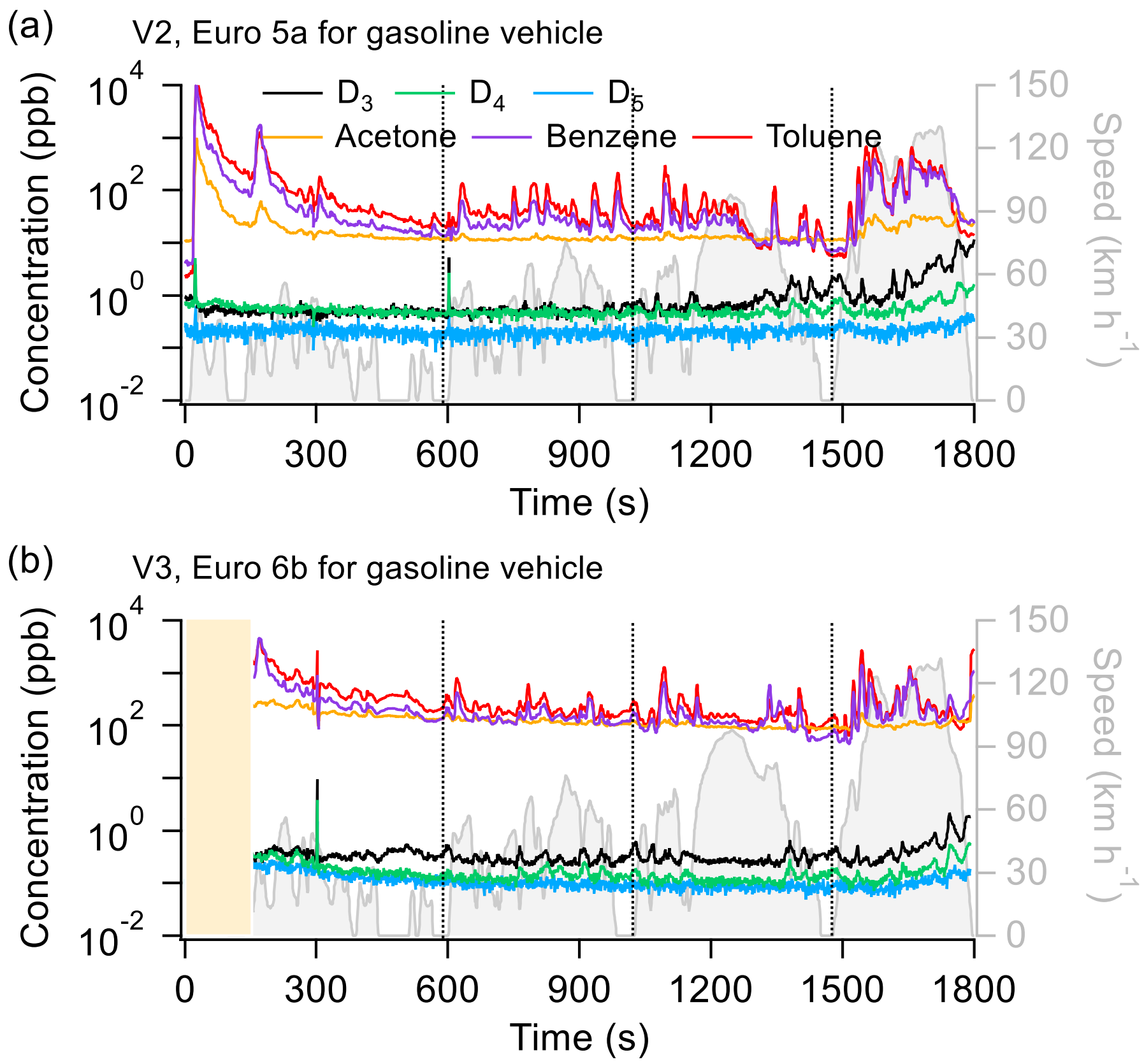

Figure S4. Time series of selected NMOG concentrations in the exhaust of light-duty gasoline vehicles with emission standard of (a) Euro 5a and (b) Euro 6b under the cold-start conditions during the WLTC cycle. Gray shaded areas indicate the speed of the vehicles on the chassis dynamometer. The concentrations in the figures are dilution-corrected values.

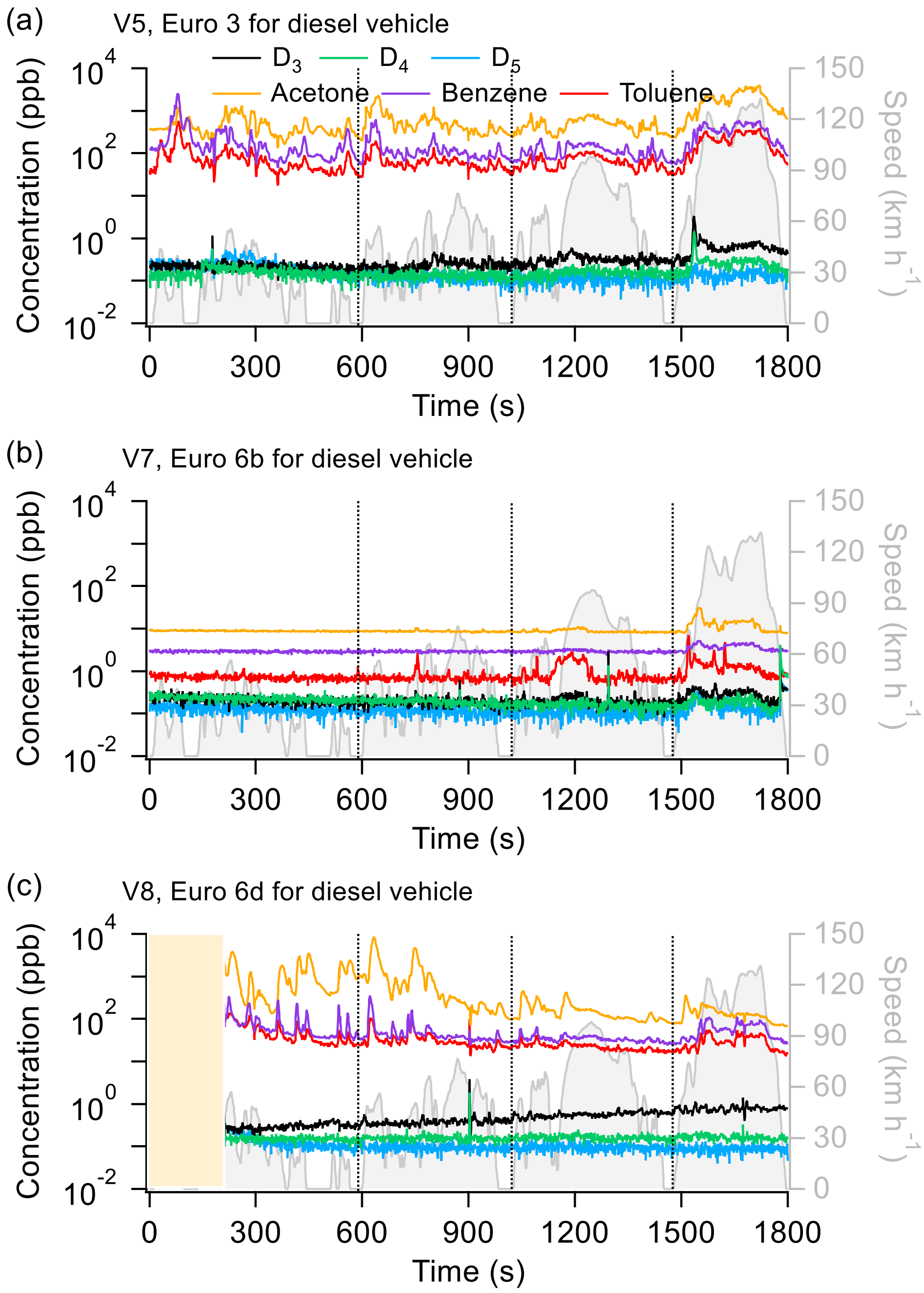

Figure S5. Time series of selected NMOG concentrations in the exhaust of light-duty diesel vehicles with emission standard of (a) Euro 3, (b) Euro 6b and (c) Euro 6d under the cold-start conditions during the WLTC cycle. Gray shaded areas indicate the speed of the vehicles on the chassis dynamometer. The concentrations in the figures are dilution-corrected values.

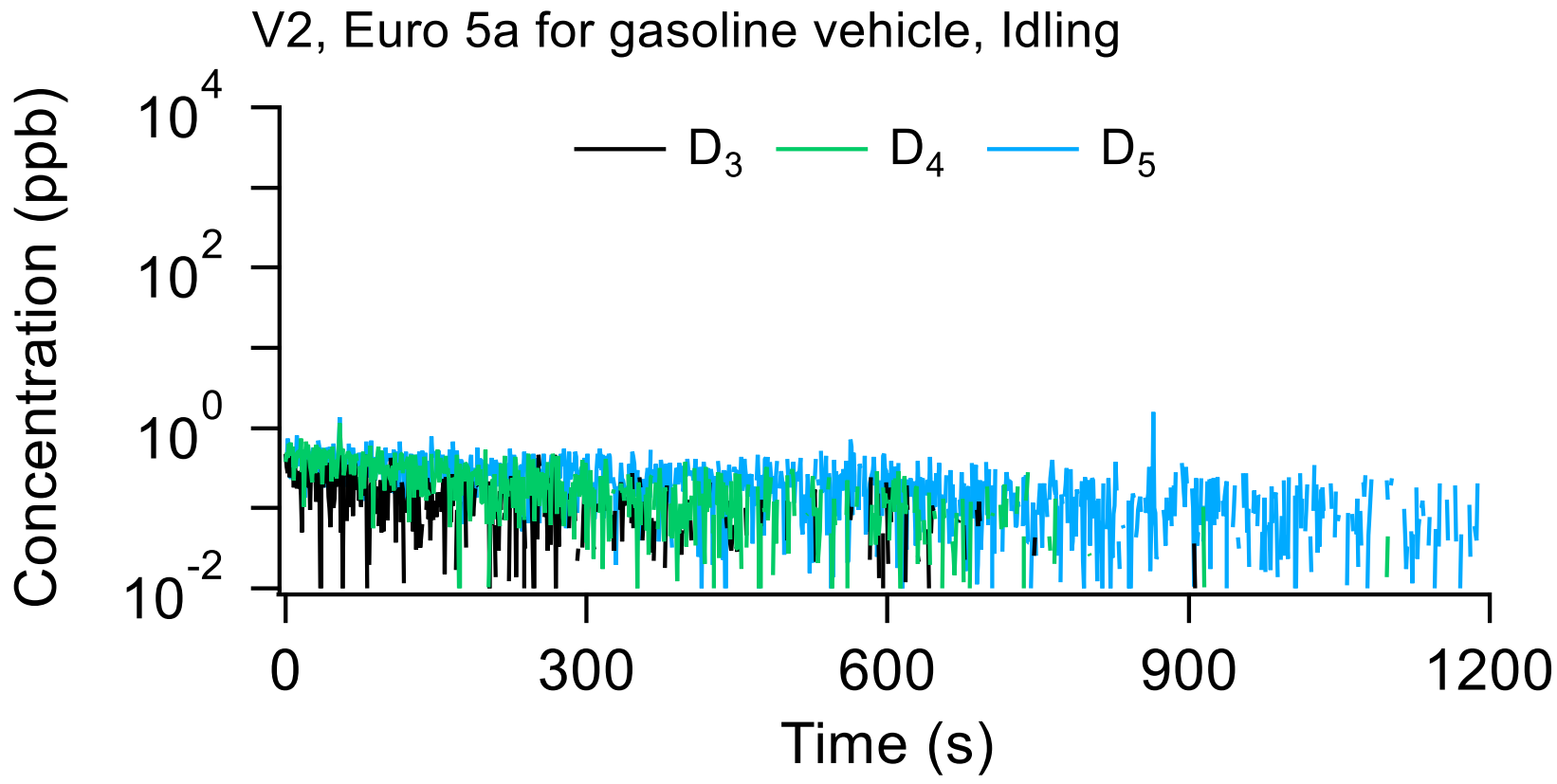

Figure S6. Time series of selected VMS concentrations in the exhaust of a Euro 5a light-duty gasoline vehicle during idling. The concentrations in the figures are dilution-corrected values.

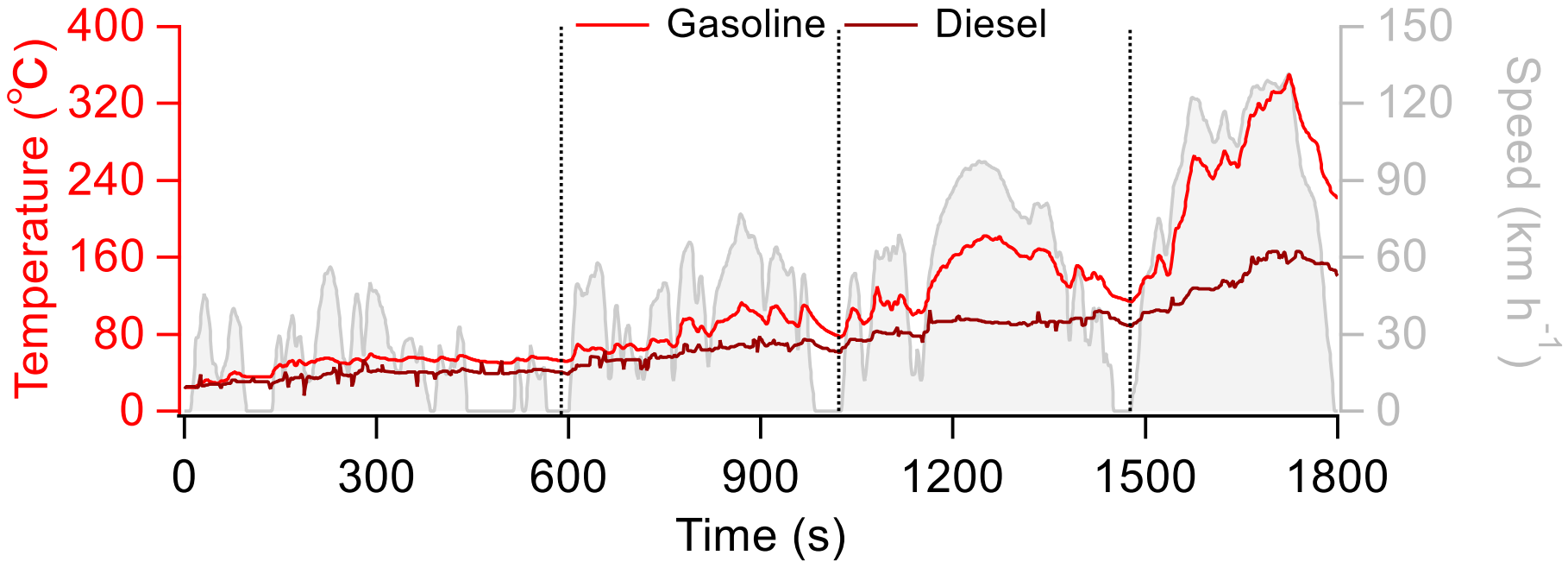

Figure S7. Time series of the exhaust temperature under the cold-start conditions during the WLTC cycle. Gray shaded areas indicate the speed of the vehicles on the chassis dynamometer.

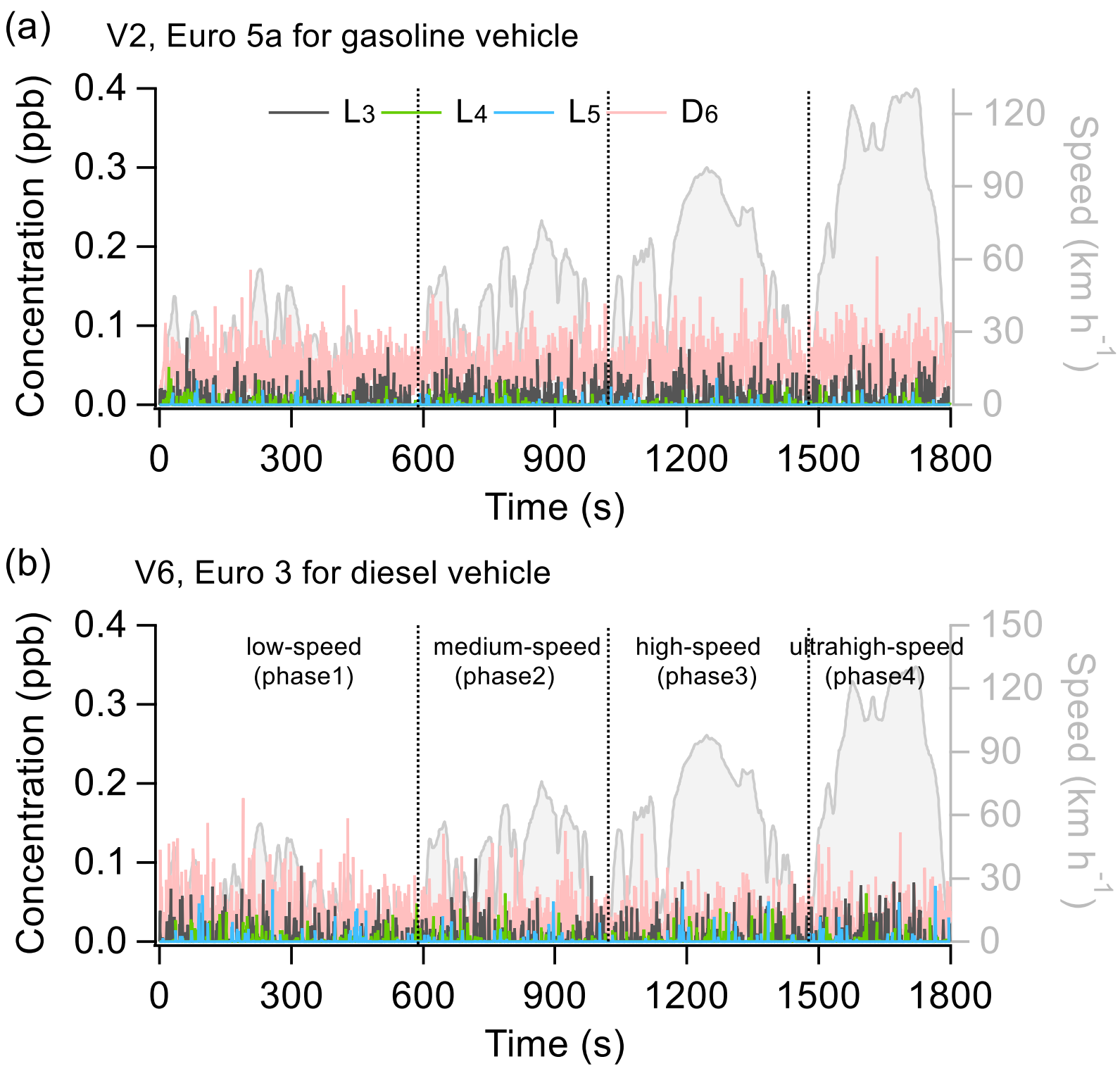

Figure S8. Time series of selected VMS concentrations in the exhaust of (a) a Euro 5a light-duty gasoline vehicle and (b) a Euro 3 light-duty diesel vehicle under the cold-start conditions during the WLTC cycle. Gray shaded areas indicate the speed of the vehicles on the chassis dynamometer. The concentrations in the figures are dilution-corrected values.

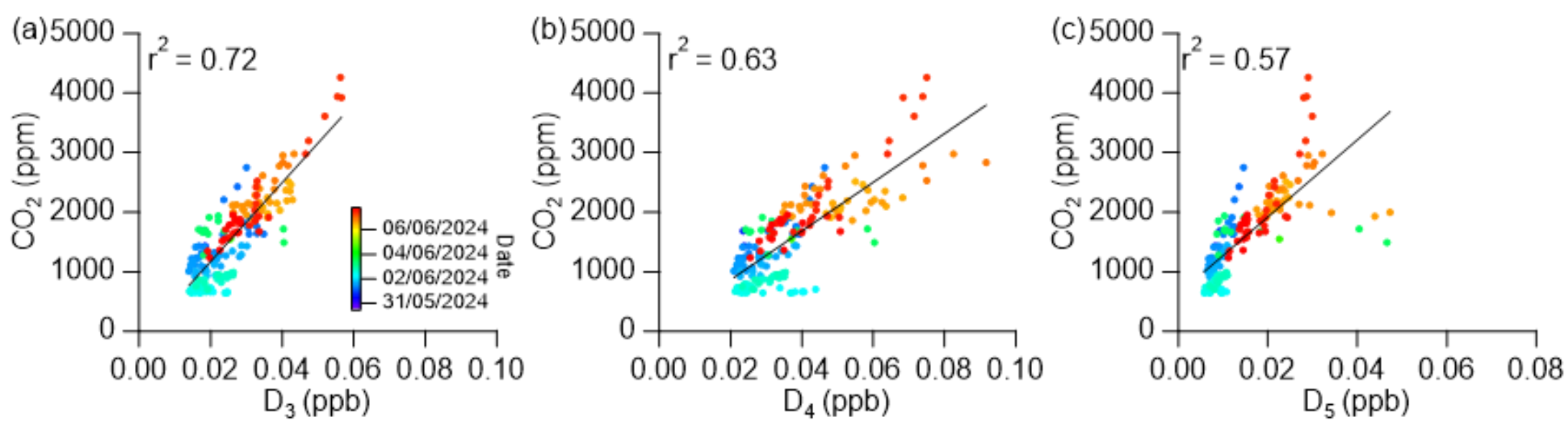

Figure S9. Scatter plot of CO_2 and VMS species measured in the tunnel. The data were averaged to 15 minutes.

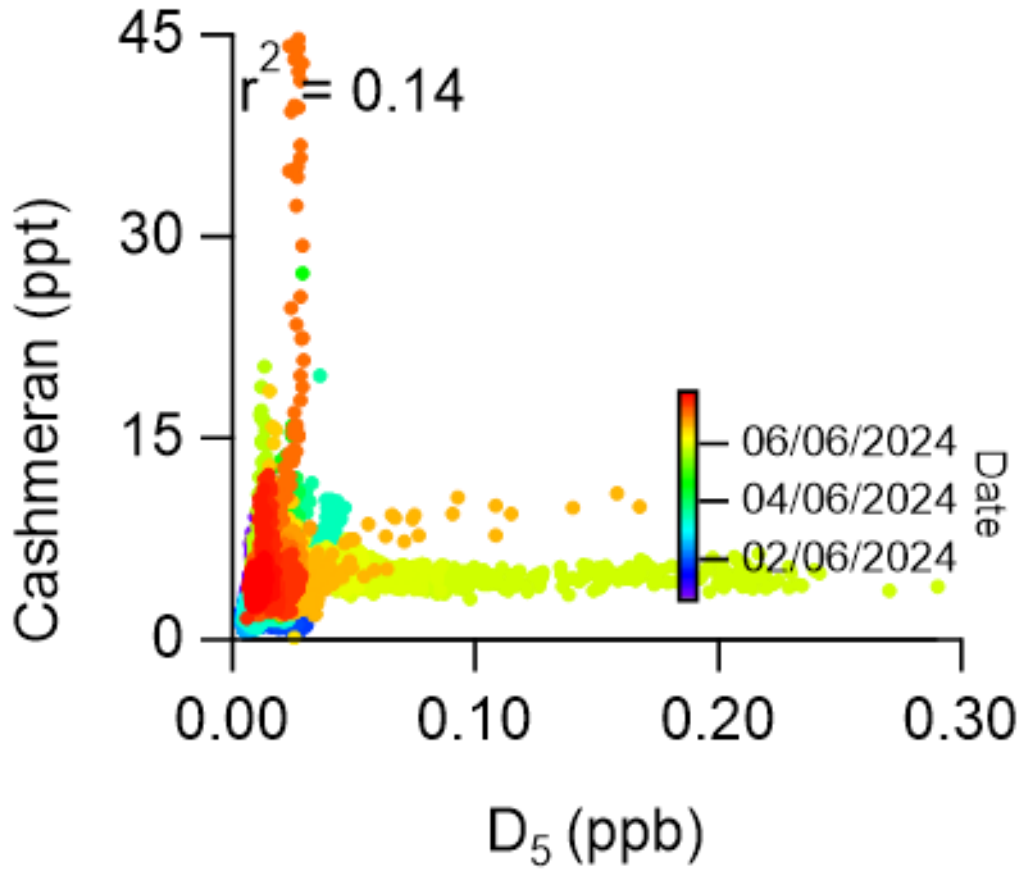

Figure S10. Scatter plot of cashmeran and D_5 measured in the tunnel. The data were averaged to 10 seconds.

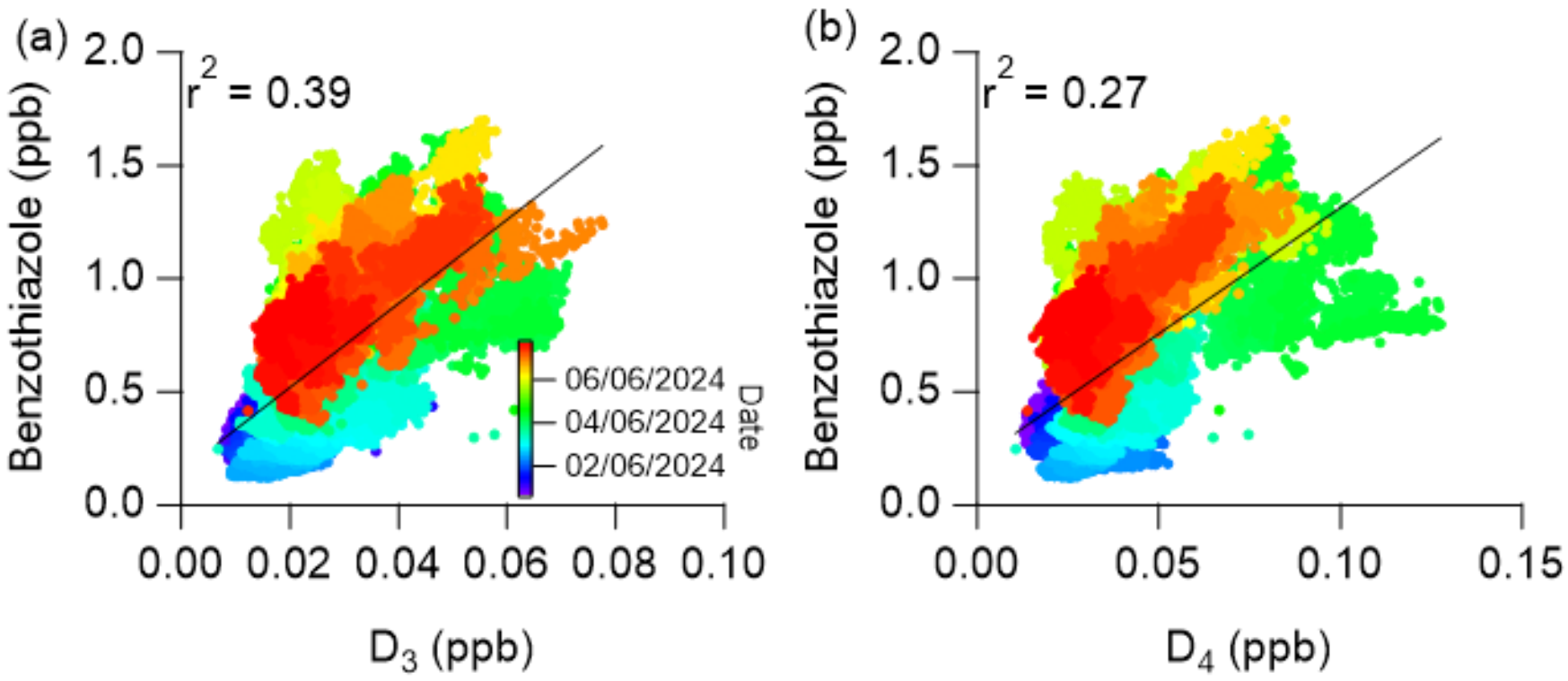

Figure S11. Scatter plot of benzothiazole and VMS species measured in the tunnel. The data were averaged to 10 seconds.

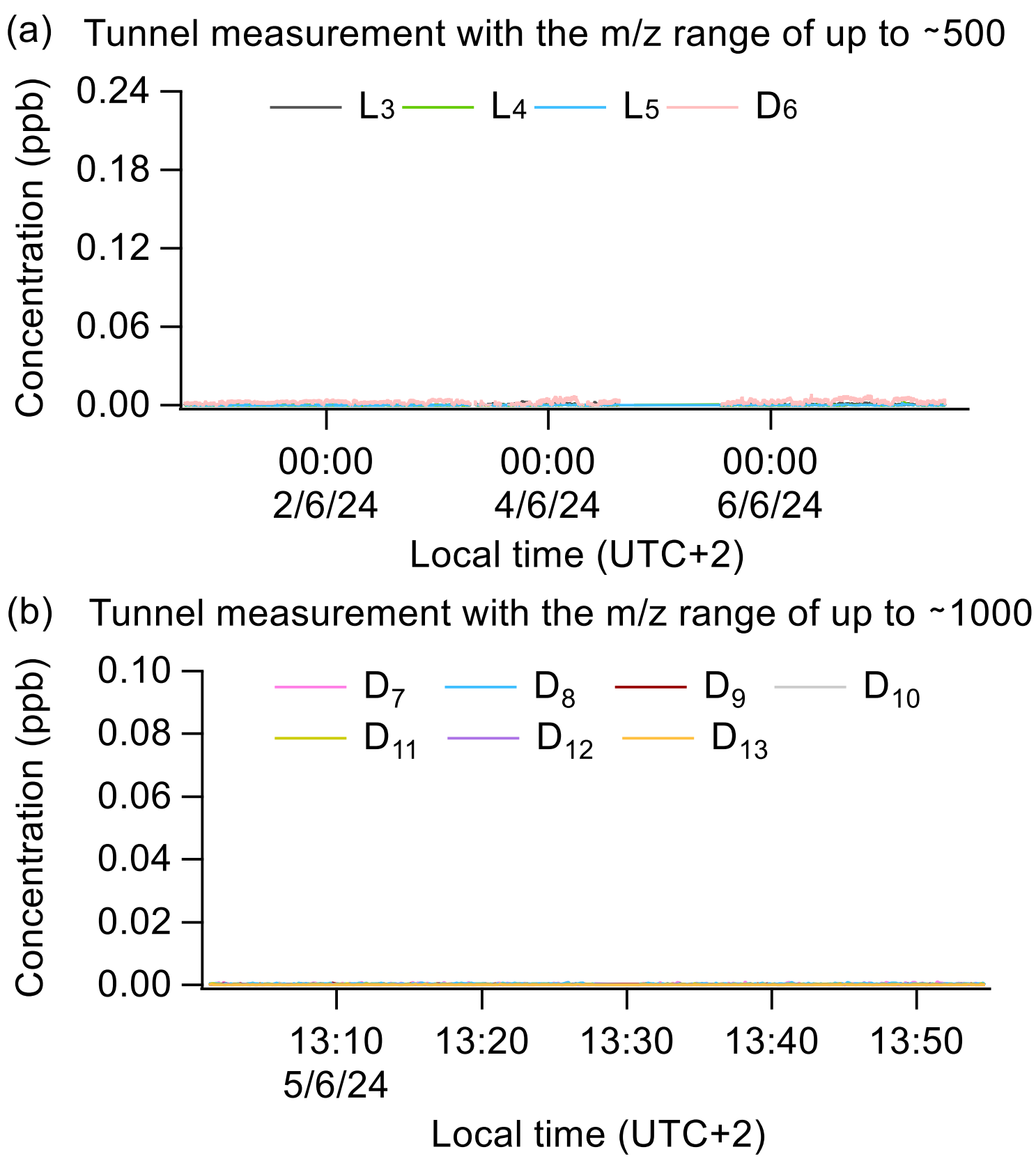

Figure S12. Time series of representative linear and cyclic VMS in the tunnel for (a) m/z < 500 and (b) 500< m/z < 1000.

Table S1. Detailed information of the vehicles used in the chassis dynamometer tests. Due to the technical issue on the vehicle, the data corresponding to V1 is not included in this study.

ID	Test Date	Model Year	Fuel	Aftertreatment	Emission Standard	Mileage (km)
V1	13/03/2024	2008	gasoline	TWC	Euro 4	138,437
V2	02/04/2024	2012	gasoline	TWC	Euro 5a	57,628
V3	19/03/2024	2015	gasoline	TWC	Euro 6b	78,755
V4	22/03/2024	2023	gasoline	TWC, GPF	Euro 6d	18,404
V5	27/03/2024	2004	diesel	DOC	Euro 3	116,419
V6	03/04/2024	2003	diesel	DOC	Euro 3	72,330
V7	04/04/2024	2015	diesel	DOC, DPF, SCR	Euro 6b	64,723
V8	26/03/2024	2021	diesel	DOC, DPF, SCR	Euro 6d	55,874

Reference:

(1) Platt, S. M.; El Haddad, I.; Pieber, S. M.; Zardini, A. A.; Suarez-Bertoa, R.; Clairotte, M.; Daellenbach, K. R.; Huang, R. J.; Slowik, J. G.; Hellebust, S.; et al. Gasoline cars produce more carbonaceous particulate matter than modern filter-equipped diesel cars. *Sci. Rep.* **2017**, *7* (1), 4926. DOI: 10.1038/s41598-017-03714-9.
